# From anaphylaxis to eosinophilic esophagitis during omalizumab treatment in a boy with severe asthma and food allergy: a case report

**DOI:** 10.3389/fimmu.2026.1836384

**Published:** 2026-05-29

**Authors:** Urszula Jedynak-Wąsowicz, Liliana Klim, Ewa Cichocka-Jarosz

**Affiliations:** 1 Clinic of Pediatrics, Jagiellonian University Medical College, Krakow, Poland; 2 Department of Pulmonology, Allergy and Dermatology, Children’s University Hospital, Krakow, Poland; 3 Students' Scientific Group (SSG) at Children’s Diseases Clinic, Children’s University Hospital, Jagiellonian University Medical College, Krakow, Poland

**Keywords:** anaphylaxis, anti-IgE therapy, atopic march, cow’s milk allergy, eosinophilic esophagitis

## Abstract

**Background:**

Eosinophilic esophagitis (EoE) is a chronic inflammatory disease of the esophagus with symptoms of esophageal dysfunction. It is strongly associated with other atopic conditions in patients and has therefore been recognized as a late manifestation of the atopic march. Sensitization to foods and/or aeroallergens early in life may predispose some individuals to subsequent EoE development. Moreover, the disease was found to emerge as a potential side effect of oral immunotherapy (OIT). However, it remains unclear whether OIT induces or rather “unmasks” a pre-existing but subclinical disorder.

**Case presentation:**

We report a boy with IgE-mediated cow’s milk (CM) allergy, confirmed in infancy after anaphylaxis to yogurt, positive skin prick tests, and elevated CM-specific IgE. He tolerated baked milk following a supervised oral food challenge. From early childhood, he developed severe allergic asthma and rhinitis, refractory to standard therapy. Omalizumab (OMA) was initiated at 10 years of age with marked and sustained asthma control. While receiving anti-IgE treatment, at 13 years of age, the patient independently liberalized his diet and tolerated progressively less processed dairy products for 6–8 months without immediate hypersensitivity reactions. He subsequently developed recurrent vomiting and, two months later, experienced anaphylaxis after ingestion of a small amount of ice cream. Despite extensive evaluation during hospitalization, vomiting persisted and was associated with weight loss. Upper endoscopy revealed esophageal inflammation, with histological confirmation of EoE diagnosis. Treatment with proton pump inhibitor therapy, swallowed topical budesonide, and renewed strict CM elimination resulted in rapid clinical improvement, weight gain, reduction of peripheral eosinophilia, and histologic remission. Treatment with OMA was sustained.

**Conclusions:**

This case illustrates the diagnosis of eosinophilic esophagitis in a child with severe IgE-mediated cow’s milk allergy and asthma during ongoing omalizumab treatment and prolonged unsupervised reintroduction of cow’s milk products. Causal inferences cannot be made, the case highlights the need to consider EoE in highly atopic patients who develop persistent upper gastrointestinal symptoms during dietary liberalization.

## 1 Introduction

Eosinophilic esophagitis (EoE) is a chronic inflammatory disease of the esophagus characterized by symptoms of esophageal dysfunction and a predominant eosinophilic infiltration of the squamous epithelium on histologic examination (1). It is a complex immunologic disorder, with antigen-driven type 2 inflammation (2). Clinical manifestations comprise dysphagia, vomiting, nausea, food impactions, chest pain and other symptoms of esophageal dysfunction (3). Established risk factors for EoE are atopy and allergic conditions, including asthma, allergic rhinitis, atopic dermatitis and food allergy, with high incidence rates in patients suffering from this disorder (3–5). EoE has also been recognized as a late manifestation of the atopic march, as sensitization to foods and/or aeroallergens early in life may predispose some individuals to subsequent EoE development (6, 7). Finally, EoE has emerged as a side effect of oral immunotherapy (OIT), primarily to food allergens, but also to inhalant allergens in some patients (2, 8, 9). Diagnosis of EoE during OIT is an indication for termination of desensitization (8). However, it remains unclear whether the OIT induces EoE in patients undergoing treatment or rather “unmasks” pre-existing but subclinical disease (10).

This case report presents a unique clinical scenario: a child with life-threatening IgE-mediated cow’s milk allergy who appeared to tolerate progressively less processed cow’s milk products while receiving omalizumab for severe asthma, and was subsequently diagnosed with histologically confirmed EoE. This case bridges pediatric allergy, pulmonology, and gastroenterology, offering insights relevant to multiple specialties managing atopic children and also underscoring the importance of multidisciplinary approach.

## 2 Case presentation

We describe the case of a boy with cow’s milk (CM) allergy, born at term as a healthy neonate and breastfed until 9 months of age. His mother followed a CM-free diet during breastfeeding due to the child’s atopic dermatitis. At the age of 6 months, the patient experienced an anaphylactic reaction after yogurt ingestion. Cow’s milk allergy was confirmed by positive skin prick tests and elevated CM-specific IgE. A strict CM elimination diet was implemented. Over time, tolerance to baked milk (32 mL) was confirmed by a supervised oral food challenge.

From early childhood, at the age of 4 years, the patient developed allergic asthma and allergic rhinitis, requiring regular treatment with inhaled corticosteroids, leukotriene receptor antagonists, antihistamines (2^nd^ gen nsAH1) and intranasal corticosteroids. Despite stepwise escalation of therapy, asthma control progressively worsened, with frequent exacerbations and several hospitalizations. At the age of 10 years, treatment with omalizumab (Xolair, 450 mg every 2 weeks) was initiated, resulting in marked and sustained improvement in asthma control and a significant reduction in exacerbations.

While receiving omalizumab, beginning at the age of 13 years, the patient gradually and independently liberalized his diet. Initial sIgE levels for CM extract and casein were 96.6 kU/L and 22.8 kU/L, respectively. He introduced subsequently less processed dairy products than baked milk, including yogurt, cheese, pizza, and pancakes, all of which were tolerated without immediate allergic symptoms. Over the following 6–8 months, he regularly (almost every day) consumed predominantly smoked cheese, without immediate hypersensitivity reactions. Recurrent daily vomiting then developed, followed approximately 2 months later by a breakthrough anaphylactic reaction after ingestion of approximately 15 mL of dairy ice cream. Symptoms occurred 5 minutes after ingestion and included vomiting, abdominal cramps, diarrhea, urticaria, dyspnea, and wheezing. The reaction occurred outside the hospital setting, and intramuscular epinephrine was not administered by the caregivers. Instead, a double dose of oral antihistamines and inhaled budesonide/formoterol were given, which led to complete symptom resolution. Because the episode fulfilled the clinical criteria for anaphylaxis, intramuscular epinephrine would have been the recommended first-line treatment according to current guidelines.

The concentration of sIgE levels for CM extract and casein were 13.3 kU/L and 3.2 kU/L, respectively. At that time, the patient’s medications included omalizumab, inhaled budesonide/formoterol, rupatadine, and vitamin D_3_.

**Figure 1:**
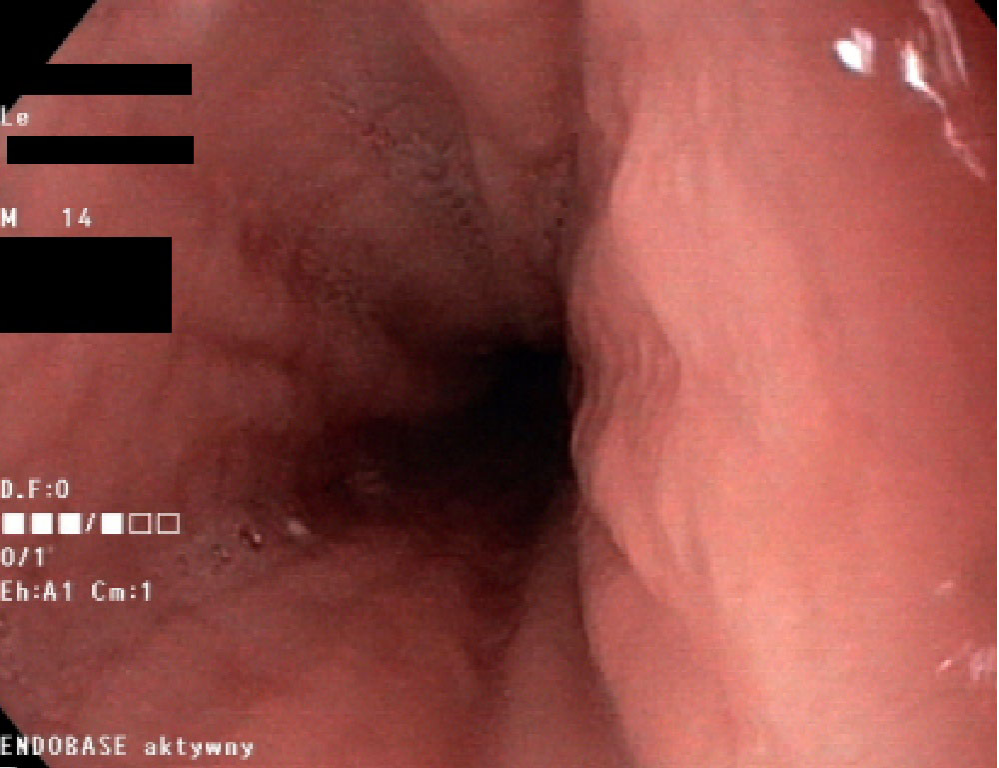
Endoscopic findings in the esophagus: furrows and edema.

**Figure 2:**
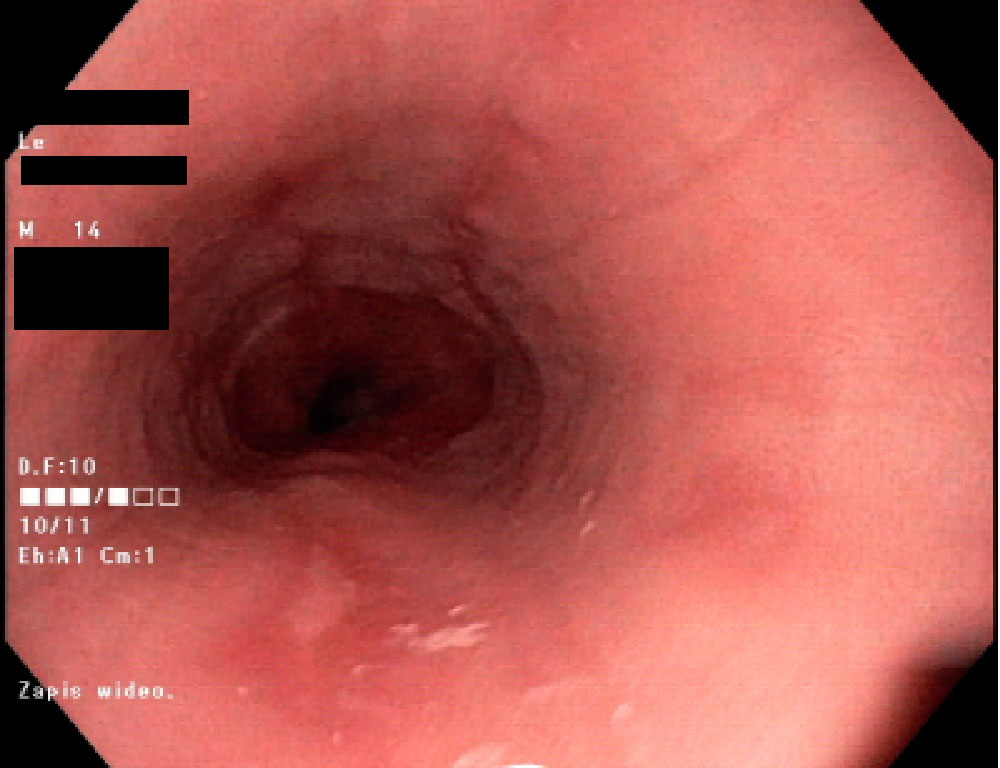
Endoscopic findings in the esophagus: rings and exudate.

Due to persistent vomiting, the patient was hospitalized. Physical examination on admission was unremarkable. Laboratory investigations showed no signs of systemic inflammation, anemia, or biochemical abnormalities. Peripheral eosinophil count was 700/μL. Stool tests for *Helicobacter pylori*, parasites, and occult blood were negative. The diagnostic work-up also considered infectious, structural, neurologic, and functional causes of persistent vomiting. Abdominal ultrasonography and contrast esophagography revealed no abnormalities. Brain magnetic resonance imaging was also normal. Despite extensive diagnostic evaluation, vomiting persisted throughout hospitalization, resulting in a weight loss of 2.5 kg over 9 days. Owing to depressive symptoms related to his father’s neoplastic disease, fluoxetine therapy was initiated under psychiatric supervision. Upper gastrointestinal endoscopy demonstrated hypertrophic and inflamed esophageal mucosa with linear erosions and thickened folds (Edema/Rings/Exudate/Furrows/Stricture score: 4), as presented in **Figures 1** and **2**.

Multiple esophageal biopsies (4 biopsies: antrum, 38 cm, 33 cm, 28 cm measured from the upper incisors) were obtained from the proximal and distal esophagus. Histopathology revealed up to 27 eosinophils per high-power field, with an overall EoEHSS (Eospinophilic Esophagitis Histologic Scoring System) score of 8 points. These findings correspond to active eosinophilic esophagitis, characterized by ongoing inflammatory changes and early structural remodeling.

**Figure 3:**
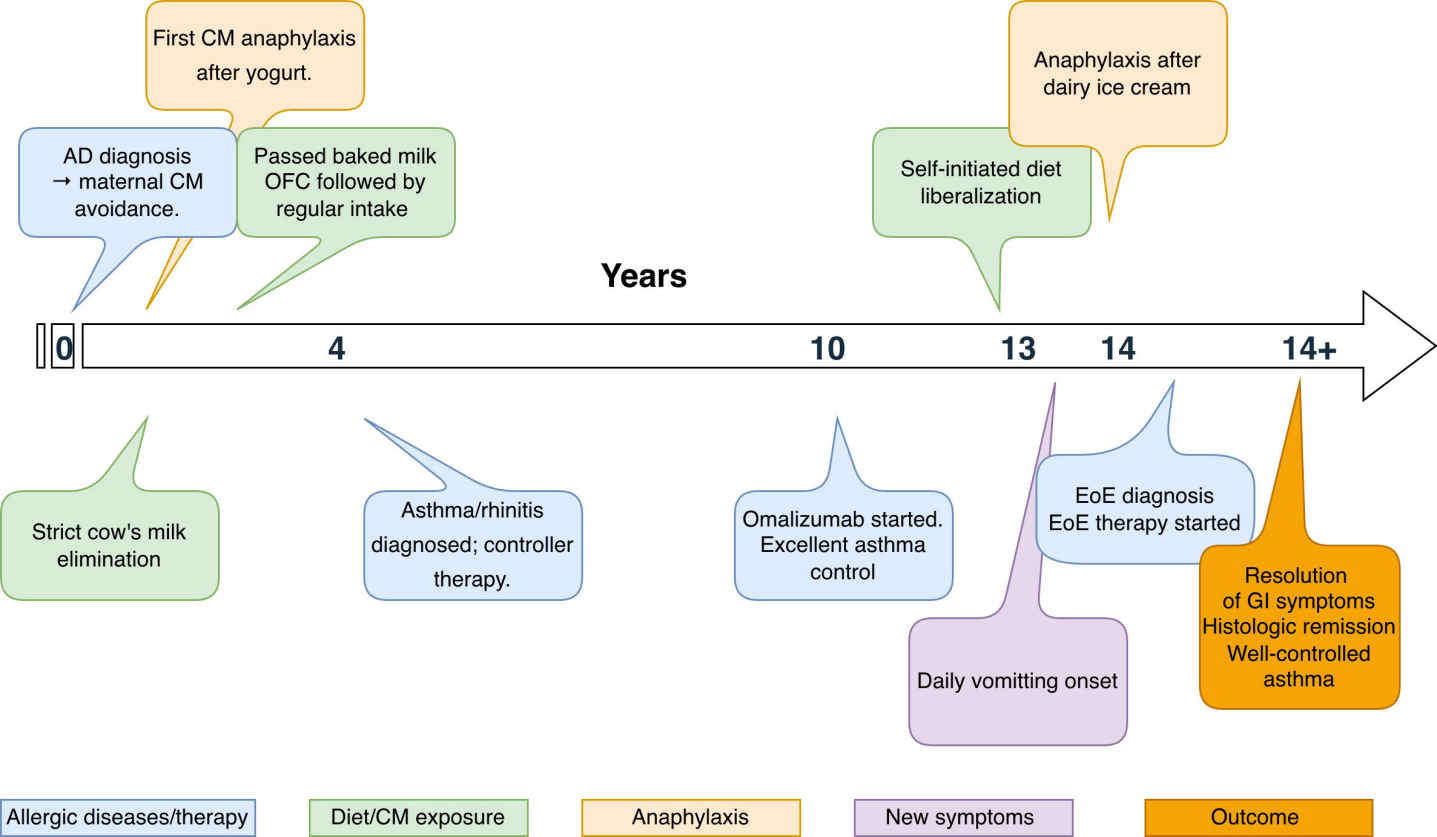
Patient’s symptoms and disease timeline.

Treatment with proton pump inhibitor therapy (60 mg/day) and swallowed topical budesonide (1000 µg/day) was initiated together with renewed strict cow’s milk elimination after endoscopy, not before. This treatment regimen was maintained for 10 weeks, until follow-up endoscopy (in accordance with current recommendations). The patient tolerated treatment well, with rapid clinical improvement, weight gain, and subsequent histologic remission. Omalizumab was continued because asthma control remained excellent, and no evidence suggested that discontinuation would be beneficial for EoE management. Peripheral blood eosinophil count decreased to 200/μL. Follow-up endoscopy demonstrated marked regression of esophageal inflammation, with a maximum of 6 eosinophils per high-power field. The PPI dose was subsequently reduced to 40 mg/day, swallowed budesonide was discontinued, and a strict CM-free diet was maintained. A summary of our patient’s symptoms and disease timeline is presented in **Figure 3**.

## 3 Discussion

This case illustrates the coexistence over time of different allergic disease phenotypes in the same patient, including early IgE-mediated cow’s milk allergy and later eosinophilic esophagitis, a predominantly antigen-driven type 2 inflammatory disorder with distinct immunopathogenic mechanisms. Despite effective suppression of IgE-dependent pathways with anti-IgE therapy, the patient developed eosinophilic esophagitis (EoE), highlighting distinct immunopathogenic mechanisms underlying IgE-mediated food allergy and eosinophilic gastrointestinal disease (3).

Cow’s milk (CM) allergy is a well-recognized risk factor for EoE, and food antigens act as disease triggers primarily through antigen-specific T-cell responses rather than classical IgE-mediated mechanisms (11). In contrast to IgE-mediated food allergy, EoE pathogenesis is driven by Th2 cytokines such as interleukin-5 and interleukin-13, leading to eosinophil recruitment and esophageal tissue inflammation. These pathways are largely unaffected by anti-IgE therapy (2, 3).

From a mechanistic perspective, omalizumab effectively reduces free IgE levels and attenuates IgE-mediated effector-cell activation, thereby increasing the threshold for immediate hypersensitivity reactions (12, 13). In the United States, it has been approved by the Food and Drug Administration (FDA) for use in patients with severe multiple food allergy to reduce the risk of anaphylaxis, especially after accidental exposure (12, 14). However, eosinophilic esophagitis is primarily driven by antigen-specific T-cell responses and type 2 cytokine pathways, especially interleukin-5 and interleukin-13, rather than by classical IgE-mediated mechanisms. Therefore, anti-IgE therapy would not be expected to prevent EoE development directly. In such a setting, renewed exposure to the culprit food during apparent clinical tolerance may coincide with the recognition of an underlying eosinophilic disorder that had previously been subclinical. Nevertheless, this case does not allow distinction between *de novo* disease development and unmasking of pre-existing EoE (15). Similar observations of EoE diagnosed during omalizumab treatment have been reported in the literature, although such reports do not establish causality (9, 16).

The patient’s tolerance of dairy products for several months without immediate hypersensitivity reactions supports the concept that suppression of IgE-mediated effector pathways may increase the clinical threshold for allergic reactions without inducing true immunological tolerance and therefore does not permit patients to consume the culprit food with impunity (12). The subsequent onset of chronic gastrointestinal symptoms and histologically confirmed EoE shows that apparent tolerance to dairy products during anti-IgE therapy did not exclude later recognition of eosinophilic esophagitis in this patient.

The patient’s history also fits well within the concept of the atopic march. He initially presented with atopic dermatitis in infancy, followed by IgE-mediated food allergy, asthma and allergic rhinitis, and eventually EoE, which is increasingly recognized as a “late” manifestation within the atopic spectrum (6, 7).

The diagnostic process in this case also underscores the challenges of recognizing EoE in patients with complex atopic disease. The boy’s vomiting initially occurred without abdominal pain, dysphagia or weight loss and was overshadowed by his history of severe asthma and food allergy. Because there were no objective signs of organic gastrointestinal disease at that time, and in the context of coexisting depressive symptoms, we initially considered functional vomiting secondary to an underlying mood disorder as the most likely explanation. Extensive evaluation for infectious, structural and neurologic causes, which is essential in management of prolonged vomiting in pediatric patients (17), was unrevealing. Only endoscopic assessment with biopsy provided the correct diagnosis. This emphasizes that persistent upper gastrointestinal symptoms in patients with food allergy—especially in the setting of recent changes in diet or immunomodulatory therapy—should prompt consideration of EoE and timely referral for endoscopy, which is considered gold standard for diagnosis and follow-up of EoE (1).

Therapeutically, our patient responded well to a combination of high-dose proton-pump inhibitor, swallowed topical corticosteroid and renewed elimination of CM, with rapid improvement in symptoms, weight gain and marked histologic remission. Asthma control remained good on continued omalizumab. This supports current concepts that EoE and IgE-mediated food allergy, although often co-existing and triggered by the same foods, represent overlapping but distinct immunologic pathways that may require separate but complementary treatment strategies (3).

An additional clinically relevant aspect of this case is the management of the breakthrough anaphylactic reaction. Although the episode fulfilled clinical criteria for anaphylaxis, intramuscular epinephrine was not administered, and symptom resolution was achieved after oral antihistamines and inhaled bronchodilator therapy. This reflects real-life caregiver management rather than recommended treatment. Current guidelines clearly indicate intramuscular epinephrine as the first-line therapy for anaphylaxis, and this case underlines the continued need for education of patients and families regarding prompt recognition and appropriate emergency treatment.

From a clinical practice perspective, this case raises several important points. First, children with severe IgE-mediated CM allergy who receive biologics such as omalizumab may experience apparent improvement in their food tolerance, but any decision to liberalize the diet should be made under specialist supervision with clear protocols and regular follow-up (12, 18). Unsupervised reintroduction of highly allergenic foods carries a risk of breakthrough immediate reactions and may complicate the interpretation of newly emerging gastrointestinal symptoms. In such situations, EoE should be considered in the differential diagnosis. Our case adds to the limited literature showing that apparent clinical tolerance to a food allergen during anti-IgE treatment and later diagnosis of EoE are not mutually exclusive. However, this observation does not allow any conclusion as to whether omalizumab influenced the emergence of EoE or whether a pre-existing subclinical disease was recognized during renewed allergen exposure.

It is important to distinguish this case from eosinophilic esophagitis diagnosed during oral immunotherapy, where regular allergen exposure is a defined therapeutic intervention. In our patient, there was no formal desensitization protocol. Instead, the clinical scenario involved unsupervised reintroduction of progressively less processed cow’s milk products during ongoing omalizumab therapy for severe asthma. Therefore, the present report should not be interpreted as evidence of omalizumab-induced EoE, but rather as a descriptive observation of EoE recognized during this clinical course.

## 4 Study strengths and limitations

This case has several strengths. It presents the evolution of allergic disease in a single patient, from IgE-mediated cow’s milk allergy and atopic dermatitis in early life, through severe allergic asthma requiring biologic treatment, to eosinophilic esophagitis diagnosed in adolescence. This clinical course is consistent with the atopic march and illustrates the overlap of different allergic phenotypes in one individual.

The diagnosis of EoE was well documented, with both endoscopic and histopathologic confirmation. Disease severity was evaluated using established measures (EREFS and EoEHSS), and follow-up endoscopy showed improvement after treatment.

The case is also strengthened by its multidisciplinary context, bringing together pediatric allergy, pulmonology, gastroenterology, and psychiatry. This reflects the reality of managing complex atopic disease in clinical practice.

The report also raises an important clinical question about clinical coexistence of anti-IgE treatment, apparent increase in clinical reactivity threshold to cow’s milk, and later diagnosis of EoE.

This case also has several limitations. As a single-patient observation, it does not allow any causal conclusions about whether omalizumab contributed to the development of EoE, unmasked a pre-existing condition, or was not related to it. It is also not possible to determine with certainty whether EoE developed *de novo* after dairy reintroduction or whether a subclinical disease process had already been present. Because endoscopy had not been performed before cow’s milk was reintroduced into the diet, baseline esophageal histology was not available.

Another limitation is the presence of psychological distress and the initiation of fluoxetine during the diagnostic work-up, which initially made the interpretation of persistent vomiting more difficult. However, the final diagnosis was supported by objective histopathologic findings and by a clear clinical and endoscopic response to EoE-directed treatment.

In addition, the report does not allow the relative contribution of each treatment component to be determined. Proton pump inhibitor therapy, swallowed topical corticosteroids, and renewed cow’s milk elimination were introduced at the same time, so their individual effects cannot be separated.

An additional limitation is the lack of baseline endoscopic or histologic assessment before dietary liberalization, as upper gastrointestinal endoscopy is not routinely performed in asymptomatic children with food allergy in the absence of suggestive gastrointestinal symptoms. Therefore, pre-existing subclinical EoE cannot be excluded.

## 5 Conclusion

In conclusion, this case describes the diagnosis of eosinophilic esophagitis in a child with severe IgE-mediated cow’s milk allergy and asthma during ongoing omalizumab treatment and prolonged unsupervised reintroduction of cow’s milk products. Although causal inferences cannot be made, the case highlights the need to consider EoE in highly atopic patients who develop persistent upper gastrointestinal symptoms during dietary liberalization.

## 6 Patient perspective

Before these stomach problems started, Xolair made a big difference in my life. My asthma got much better, and I was able to play football, which is my favorite sport, almost every day. I also won long-distance running competitions, and that meant a lot to me. After some time, I noticed that if I accidentally ate small amounts of dairy products, at first more processed ones (as my Doctor allowed me) and later even less processed ones, I did not have allergic reactions. That made me feel more normal, like the other friends at school.

Later, when I started experiencing vomiting and feeling worse, it was very confusing and upsetting for me and my family. It was hard to understand why this was happening, especially because it seemed like my milk allergy had improved. It was also a difficult time for us because my father was seriously ill. When the doctors finally found out that I had eosinophilic esophagitis, it helped me understand what was going on. After treatment started, I slowly began to feel better, my symptoms improved. This experience taught me that even when allergic reactions seem to disappear, it does not always mean that everything is fine. Now I know that it is very important to listen to the doctors, follow their advice, and not ignore new symptoms.

## Data Availability

The raw data supporting the conclusions of this article will be made available by the authors, without undue reservation.
